# Evolutionary engineering of *Lactobacillus bulgaricus* reduces enzyme usage and enhances conversion of lignocellulosics to D-lactic acid by simultaneous saccharification and fermentation

**DOI:** 10.1186/s13068-020-01812-x

**Published:** 2020-10-16

**Authors:** J. Vishnu Prasad, Tridweep K. Sahoo, S. Naveen, Guhan Jayaraman

**Affiliations:** 1 Bioprocess and Metabolic Engineering Laboratory, Department of Biotechnology, Bhupat and Jyoti Mehta School of Biosciences, Indian Institute of Technology Madras, Chennai, Tamil Nadu 600036 India; 2 Present Address: Indian Institute of Technology, BHU, Varanasi, India

**Keywords:** Adaptive laboratory evolution (ALE), *L. bulgaricus*, Simultaneous saccharification and fermentation (SSF), Lignocellulosic biomass (LCB), D-lactic acid (D-LA), Product to enzyme ratio (PER)

## Abstract

**Background:**

Simultaneous saccharification and fermentation (SSF) of pre-treated lignocellulosics to biofuels and other platform chemicals has long been a promising alternative to separate hydrolysis and fermentation processes. However, the disparity between the optimum conditions (temperature, pH) for fermentation and enzyme hydrolysis leads to execution of the SSF process at sub-optimal conditions, which can affect the rate of hydrolysis and cellulose conversion. The fermentation conditions could be synchronized with hydrolysis optima by carrying out the SSF at a higher temperature, but this would require a thermo-tolerant organism. Economically viable production of platform chemicals from lignocellulosic biomass (LCB) has long been stymied because of the significantly higher cost of hydrolytic enzymes. The major objective of this work is to develop an SSF strategy for D-lactic acid (D-LA) production by a thermo-tolerant organism, in which the enzyme loading could significantly be reduced without compromising on the overall conversion.

**Results:**

A thermo-tolerant strain of *Lactobacillus bulgaricus* was developed by adaptive laboratory evolution (ALE) which enabled the SSF to be performed at 45 °C with reduced enzyme usage. Despite the reduction of enzyme loading from 15 Filter Paper Unit/g_LCB_ (FPU/g_LCB_) to 5 FPU/g_LCB_, we could still achieve ~ 8% higher cellulose to D-LA conversion in batch SSF, in comparison to the conversion by separate enzymatic hydrolysis and fermentation processes at 45 °C and pH 5.5. Extending the batch SSF to SSF with pulse-feeding of 5% pre-treated biomass and 5 FPU/g_LCB_, at 12-h intervals (36th–96th h), resulted in a titer of 108 g/L D-LA and 60% conversion of cellulose to D-LA. This is one among the highest reported D-LA titers achieved from LCB.

**Conclusions:**

We have demonstrated that the SSF strategy, in conjunction with evolutionary engineering, could drastically reduce enzyme requirement and be the way forward for economical production of platform chemicals from lignocellulosics. We have shown that fed-batch SSF processes, designed with multiple pulse-feedings of the pre-treated biomass and enzyme, can be an effective way of enhancing the product concentrations.

## Background

Lactic acid (LA) is an important platform chemical with a wide range of applications in the food and cosmetic industries [1]. Lactic acid has two enantiomers, L-lactic acid and D-lactic acid, which can be blended in different ratios to achieve application-specific poly-lactic acid (PLA) [2]. Commercial production of LA is mostly achieved by fermentation of starch [3–5] or refined sugars; however, high cost of these substrates is one of the obstacles for fermentative LA to compete with synthetically produced LA. Cheap and renewable sugar substrates or substrates from waste agricultural residues are economically viable alternatives to refined sugar for LA production [1]. Recent trends in D-LA production have shifted to utilization of dairy waste [6] or renewable non-food materials, especially lignocellulosic biomass (LCB) from agro-industrial sources [7–11].

Valorization of LCB to bulk chemicals like D-LA requires a sequence of separate processes such as pretreatment of LCB, enzymatic hydrolysis of cellulose and fermentation of the hydrolysate. However, the accumulation of product (glucose) inhibits enzymatic hydrolysis during batch processes [12, 13] and, therefore, requires more enzyme usage to compensate for the end-product inhibition. Physical and temporal separation of the enzymatic hydrolysis and fermentation processes makes the whole process time-intensive and results in low productivity [1].

Simultaneous saccharification and fermentation (SSF) is an alternative process in which the enzymatic hydrolysis occurs concomitantly with microbial fermentation. SSF is advantageous because of reduced end-product inhibition of hydrolytic enzymes, low enzyme usage and higher throughput [14, 15]. Despite its techno-economic advantages over separate hydrolysis and fermentation (SHF), the major drawback with SSF is the difference in temperature optima of the hydrolytic enzymes and the fermenting microorganism. The enzyme cocktails, which are most frequently used in cellulose hydrolysis process have an optimum operating temperature of about 50 °C [16]. On the contrary, most of the microorganisms that have been used for microbial production of LA have optimum growth at 30–37 °C [17]. Unless the difference in the optima of the two processes is narrowed down, it can result in sub-optimal operation of the SSF. In addition to the high operating temperatures, the microbes employed in SSF are subjected to other extreme conditions associated with SSF such as low pH, osmotic pressure and inhibitors.

Performing SSF at a higher temperature which is optimal for enzymatic hydrolysis could arrest or reduce the microbial growth and would further lead to glucose accumulation, enzyme inhibition and eventually lead to reduced cellulose conversion. At the same time, operating SSF at the lower temperature which is optimal for bacterial growth could reduce the rate of enzymatic hydrolysis and the percentage conversion to glucose, and thus could lead to reduced titer of the final fermentation product. This would in turn require the usage of higher enzyme loading to achieve the desired cellulose conversions, titer and productivity, thus adversely affecting process economics. Cost of cellulolytic enzymes is one of the critical factors which contribute immensely to cost of production of a low-value, high-volume chemical like D-LA. The higher cost of cellulases compared to starch degrading enzymes demands the economical utilization of cellulases for the conversion of agricultural waste [1]. Techno-economic studies on second-generation ethanol production have revealed that cost of enzyme significantly affects the final cost of product [18–20].

Thus, a thermo-tolerant strain is needed to operate SSF at the temperature optimum of enzymatic hydrolysis for improved conversion. Adaptive laboratory evolution (ALE) is considered as a simple yet robust method to develop strain tolerance to any specific adverse condition [21]. There are numerous studies on *Saccharomyces cerevisiae* and ethanol production by SSF, using evolved strains tolerant to temperature [22, 23], pH [24] and other inhibitors [25]. Some studies have attempted to evolve yeast strains for LA production to leverage its property to tolerate lower pH [26]. There are also reports on L-LA production from lignocellulosic substrate by SSF using different strains evolved for improved antioxidant property [27] and efficient xylose assimilation [28]. Although enzyme loading is a critical techno-economic factor, there are not many detailed reports available on optimization or minimization of enzyme loading to achieve higher product to enzyme ratio (PER). An earlier study demonstrated that the enzyme loading can be reduced by 30% through media optimization in SSF for D-LA production [11]. Very few reports are available which have focused on the valorization of LCB for the production of D-LA by SSF (Additional file 1: Table S1). There are no detailed reports that compare the trade-off between the effect of temperature and different enzyme loadings on enzymatic saccharification. There are also no reports which demonstrate the utility of an evolved thermo-tolerant strain in minimizing the enzyme usage in SSF for D-LA production.

In current study, we have employed *Lactobacillus delbrueckii* subsp. *bulgaricus* for production of D-LA from rice-straw biomass. *L. bulgaricus* is one among the very few homo-D-lactic fermentative bacteria which produce optically pure D-LA [29]. It usually grows optimally at 37 °C, has glucose as the most preferred carbon source and is not capable of assimilating xylose [30]. Most of the previous studies on *L. bulgaricus* have reported an operational pH in the range of 5.2–6.0 [31–33]. Our preliminary study revealed that *L. bulgaricus* WT shows similar growth profiles at pH 5.5 and pH 6.0 (Additional file 1: Fig S1). Thus, the operating pH was kept at 5.5 for further SHF and SSF experiments.

Initial batch enzymatic hydrolysis experiments provided evidence on the relation between operating temperature, cellulose conversion rates and enzyme loadings. Preliminary SHF experiments using the wild-type strain at its optimal temperature of 37 °C proved that the bacteria could grow on the LCB hydrolysate (Additional file 1: Fig S2). Therefore, we developed a thermo-tolerant strain from wild-type *L. bulgaricus* (*L. bulgaricus* WT) by ALE which could grow at a higher temperature and produce D-LA from cellulose hydrolysate with the same yield and titer as obtained at the 37 °C fermentation process.

Subsequently, the thermo-tolerant *L. bulgaricus* (*L. bulgaricus* ET45) was employed in batch and fed-batch SSF at an elevated temperature of 45 °C for production of D-LA from LCB derived from rice straw. This study proves the efficacy of the thermo-tolerant strain in drastically reducing the enzyme consumption during SSF processes as well as improving the overall productivity without compromising on the cellulose conversion and product yield obtained with SHF processes.

## Results

### Batch enzymatic hydrolysis

**Fig. 1:**
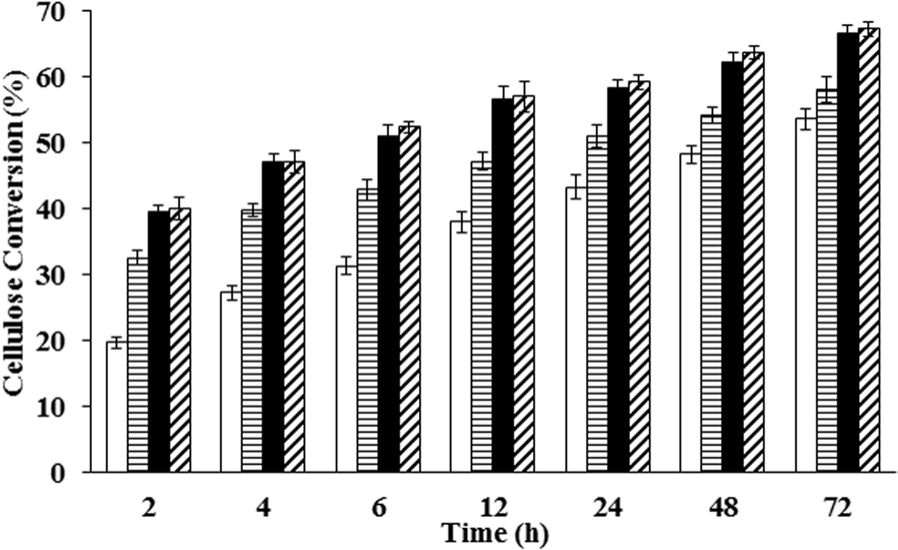
Effect of different enzyme loading on enzymatic cellulose hydrolysis. Experiment was conducted at 50 °C and pH 4.5 with 10% (w/v) pre-treated LCB (dil. acid pre-treated at 175 °C) at different enzyme loadings to evaluate cellulose hydrolysis (%) white bar-5 FPU/g_LCB_, Horizontally striped bar-10 FPU/g_LCB_, black bar-15 FPU/g_LCB_, Diagonally striped bar-30 FPU/g_LCB_ Values are mean ± SD (*n* = 3)

**Fig. 2:**
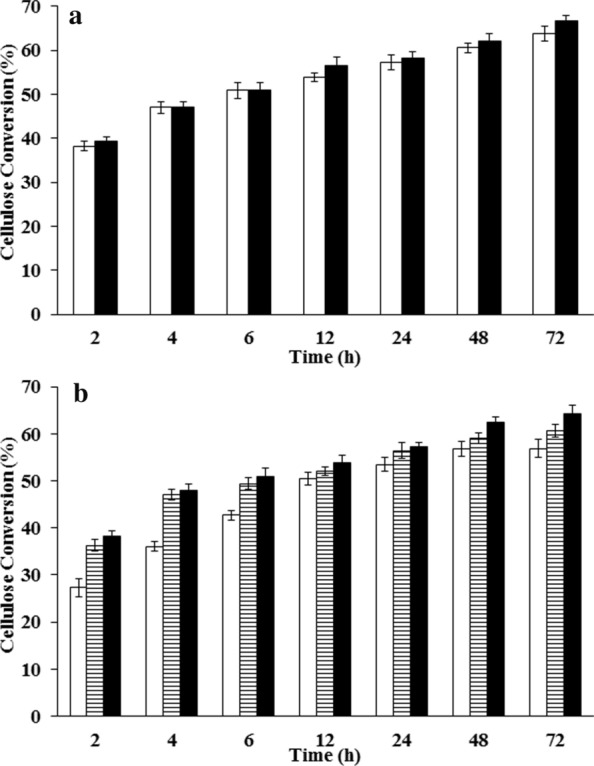
Effect of pH and temperature on enzymatic cellulose hydrolysis. Experiments are conducted with 10% (w/v) pre-treated LCB (dil. acid pre-treated at 175 °C) at 15 FPU/g_LCB_. **a** Effect of pH on cellulose hydrolysis (%) at 50 °C; white bar-pH 5.5, black bar-pH 4.5; **b** effect of temperature on cellulose hydrolysis (%) at pH 5.5; white bar-37 °C, Horizontally striped bar-45 °C, black bar-50 °C. Values are mean ± SD (*n* = 3)

The effect of enzyme loading on batch cellulose hydrolysis was studied at the optimal condition of temperature 50 °C and pH 4.5 [34–36]. It is evident from Fig. 1 that the initial hydrolysis rate and the final percentage conversion remains similar for enzyme loading beyond 15 FPU/g_LCB_. Therefore, this value (15 FPU/g_LCB_) was taken as the saturation enzyme loading for batch cellulose hydrolysis. The effect of pH was studied at the saturation enzyme loading and optimal temperature (50 °C) at pH 4.5 (optimal pH for enzymatic hydrolysis) and 5.5 (fermentation pH). There was no significant difference observed in terms of initial rate and final percentage conversion between these two pH conditions (Fig. 2a). Thus, we considered pH 5.5 as the optimal pH for SSF process, since it would be more favorable for fermentation and would not affect the enzymatic hydrolysis. The enzymatic hydrolysis was also found to be more efficient at 50 °C than at 37 °C (Fig. 2b, Additional file 1: Figs. S3, S4). Since the optimal growth temperature of most of the lactic acid bacteria is around 37 °C, a SSF process would require the use of a thermo-tolerant bacterial strain to make the process more efficient.

### Product inhibition in batch enzymatic hydrolysis

**Fig. 3:**
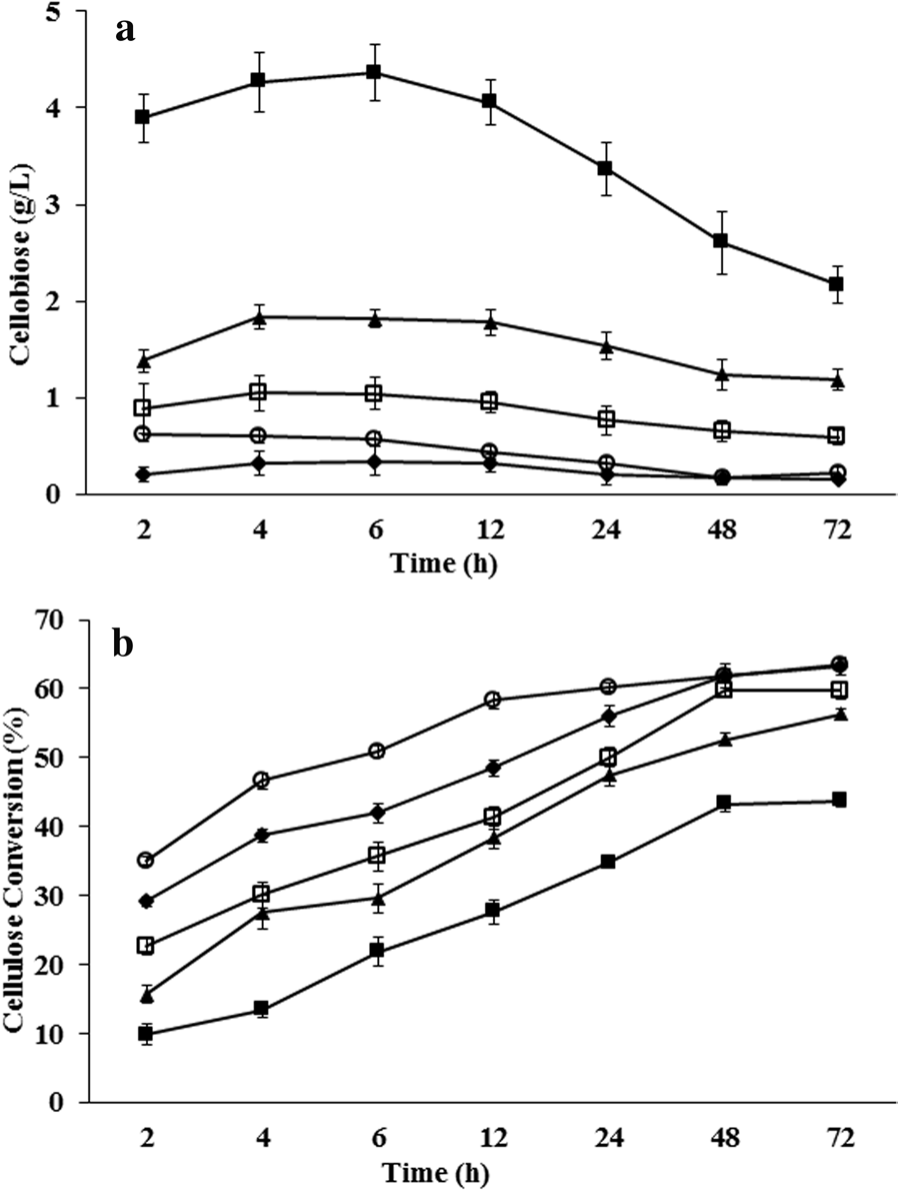
Effect of glucose on cellulose hydrolysis. Experiments were conducted at 45 °C, pH 5.5 and 10% (w/v) pre-treated LCB (dil. acid pre-treated at 175 °C) using 15 FPU/g_LCB_ Time course profile of **a** cellobiose accumulation and **b** percentage cellulose conversion with different initial concentrations of glucose externally added before the initiation of enzymatic hydrolysis. White circle—0 g/L, black diamond—5 g/L, white square—20 g/L, black triangle—40 g/L, black square—60 g/L

Product inhibition by cellobiose and glucose is a major limitation in a batch enzymatic hydrolysis of cellulose [37, 38]. LCB (pre-treated rice straw) spiked with different concentrations of glucose (0–60 g/L) or cellobiose (0–5 g/L) was subjected to batch enzymatic hydrolysis to understand the effect of product inhibition. Glucose concentration at a minimal level (≤ 5 g/L) was found to have no significant effect on the initial hydrolysis rate (during 0-2 h), cellobiose accumulation (Fig. 3a) and final percentage conversion (Fig. 3b). Glucose level beyond 20 g/L significantly inhibited the initial rate as well as the final percentage conversion. Higher initial glucose concentration (20–60 g/L) was also found to result in accumulation of cellobiose, the intermediate in conversion of cellulose to glucose (Fig. 3a). Cellobiose accumulation, in turn, inhibits the overall conversion of cellulose (Additional file 1: Fig. S5).

Therefore, removal of glucose during the hydrolysis process can result in reducing glucose and cellobiose inhibition, due to improved cellobiose utilization. This will increase the hydrolysis rates and overall conversion of cellulose to glucose. In an independent set of experiments, it was observed that removal of glucose (by intermittent replacement of supernatant with buffer) could enhance the conversion when compared to a batch hydrolysis, where the glucose was not removed (Additional file 1: Fig. S6). Furthermore, the effect of enzyme loading has significant differences only at the early stage of the hydrolysis process. As can be seen in Fig. 1, increasing the enzyme loading from 5 FPU/g_LCB_ to 15 FPU/g_LCB_ can double the hydrolysis rate within the initial 2 h, while the difference is relatively much less after 12 h of hydrolysis. Towards the late stage of hydrolysis (24–72 h), the rate of hydrolysis slows down, which could be due to combined effect of product accumulation (inhibition), decreased availability of substrate, non-productive binding of enzymes to lignin present in the acid-pre-treated LCB and partial enzyme deactivation [39, 40]. By removing the glucose during the hydrolysis, the inhibition can be reduced and the rate of hydrolysis can be improved at a lower enzyme loading (Additional file 1: Fig. S6). A similar result can be brought about by employing a SSF process, wherein the glucose formed by hydrolysis of cellulose is simultaneously converted to the fermented product. However, an optimal process condition needs to be found for the saccharification as well as fermentation.

To validate our assumption that SSF would improve the conversion efficiency because of concomitant glucose consumption by the microbe, batch SSF experiments were conducted at 37 °C and pH 5.5 and compared with separate hydrolysis and fermentation (SHF) experiments. Separate batch fermentation experiments were conducted at 37 °C with 30 g/L pure glucose to compare the growth parameters of the organism in batch fermentations with lignocellulosic hydrolysate.

### Simultaneous saccharification and fermentation by *L. bulgaricus* WT

A separate fermentation process for converting the cellulosic hydrolysate to D-LA at 37 °C and pH 5.5 gave a yield of 0.86 (g_DLA_/g_glucose_) (Table 1). The yield of cellulose to glucose by enzymatic hydrolysis at similar conditions of 37 °C and pH 5.5 using 10% solid loading (w/v) was 48.22% (Table 2). Thus the estimated conversion of cellulose to D-LA that we could have achieved in a SHF process with similar condition would have been around 41%, which is much lower than the yield we achieved in SSF process (49%) at 37 °C (Table 2). Also, we found that there is no significant difference in growth parameters when *L. bulgaricus* WT was grown on lignocellulosic hydrolysate and pure glucose (Table 1). This indicates *L. bulgaricus* WT is a suitable candidate for producing D-LA by valorization of LCBs through a biochemical route.

**Table 1:** Comparison of specific growth rate, specific glucose uptake rate and D-LA yield between *L. bulgaricus* WT and ET45

Strain	Condition	µ (h^−1^)	q_s_ (g g^−1^ h^−1^)	Y_D-LA/glucose_ (g g^−1^)
*L. bulgaricus* WT	30 g/L glucose, 37 °C, pH 5.5	0.30 ± 0.02	3.31 ± 0.2	0.86 ± 0.01
*L. bulgaricus* ET45	30 g/L glucose, 45 °C, pH 5.5	0.29 ± 0.01	3.18 ± 0.08	0.87 ± 0.01
*L. bulgaricus* WT	Hydrolysate (30 g/L glucose equivalent), 37 °C, pH 5.5	0.33 ± 0.02	3.41 ± 0.17	0.87 ± 0.02
*L. bulgaricus* ET45	Hydrolysate (30 g/L glucose equivalent), 45 °C, pH 5.5	0.32 ± 0.01	3.75 ± .05	0.86 ± 0.01

**Table 2:** Comparison of cellulose conversion in EH and SSF experiments at different conditions

Process	Total Solid loading (% w/v)	D-LA (g/L)^#^	Cellulose to D-LA conversion (%)	Cellulose to glucose conversion (%)^#^
EH 15FPU/50 °C/pH 4.5	10		57.31^#^	66.64 ± 1.23
EH 15FPU/45 °C/pH 5.5	10		52.13^#^	60.62 ± 1.43
EH 5FPU/45 °C/pH 5.5	10		43.07^#^	50.08 ± 1.05
EH 5FPU/37 °C/pH 5.5	10		41.47^#^	48.22 ± 0.71
Batch SSF (*L. bulgaricus* WT) 5FPU/37 °C/pH 5.5	5	12.54 ± 0.53	49.11	57.11^*^
Batch SSF (*L. bulgaricus* ET45) 15FPU/45 °C/pH 5.5	10	32.37 ± 0.44	63.4	73.72^*^
Batch SSF (*L. bulgaricus* ET45) 5FPU/45 °C/pH 5.5	10	30.6 ± 0.61	59.93	69.68^*^
Batch SSF (*L. bulgaricus* ET45) 5FPU/45 °C/pH 5.5	5	15.02 ± 0.33	58.83	68.41^*^
Batch SSF (*L. bulgaricus* ET45) 3FPU/45 °C/pH 5.5	5	11.82 ± 0.31	46.3	53.84^*^
Pulse-fed SSF (*L. bulgaricus* ET45) 5FPU/45 °C/pH 5.5	35	108.58 ± 1.75	60.76	70.65^*^

^#^Estimated cellulose to D-LA conversion = Cellulose to glucose conversion*Y_D-LA/Glucose_

^*^Estimated cellulose to glucose conversion = Cellulose to D-LA conversion/Y_D-LA/Glucose_

Y_D-LA/Glucose_ = 0.86, calculated from independent SHF experiments at 45 °C and pH 5.5 (Table 1)

^**#**^Measured values are mean ± SD

However, the 37 °C temperature for the SSF process was sub-optimal for enzymatic hydrolysis (Table 2). On the other hand, the fermentation could not be carried out above 40 °C due to the lack of growth of *L. bulgaricus* WT strain beyond this temperature (Additional file 1: Fig. S7). To further enhance the conversion of cellulose to D-LA, SSF needs to be carried out closer to the optimal temperature for enzymatic hydrolysis. This necessitated the generation of a thermo-tolerant strain of *L. bulgaricus* WT by ALE (as described in Materials and Methods section).

### Batch fermentation experiments with thermo-tolerant *L. bulgaricus*

Using ALE strategy, a stable thermo-tolerant strain *L. bulgaricus* ET45 was developed from the parental strain (WT). *L. bulgaricus* ET45, unlike the parental strain, is capable of growing at an elevated temperature of 45 °C (no growth was observed above 45.3 °C). Batch reactor studies using glucose (30 g/L) as substrate (Additional file 1: Fig. S8a) suggest that the specific growth rate (µ) and specific glucose uptake rate (q_s_) of ET45 strain are similar to that of WT strain (Table 1). Moreover, there was no significant change observed in D-LA yield (0.87 g/g) of *L. bulgaricus* ET45 at 45 °C as compared to WT strain at 37 °C (Table 1).

To further ascertain its growth parameters in hydrolysate, we carried out batch SHF experiment with hydrolysate containing 30 g/L glucose. In batch SHF at 45 °C, we achieved a specific growth rate and D-LA yield with the thermo-tolerant *L. bulgaricus* ET45 which was similar to that achieved with pure glucose substrate at 45 °C as well as to the parent strain grown in hydrolysate at 37 °C (Table 1, Additional file 1: Fig. S8b). Furthermore, the batch enzymatic hydrolysis experiments at 45 °C showed that there is no significant difference in cellulose conversion between 45 °C and 50 °C (Additional file 1: Fig. S4). Subsequently, SSF experiments were conducted with *L. bulgaricus* ET45 at 45 °C, and compared with the hydrolysis rate and conversion of cellulose to glucose obtained in SHF experiments.

### Simultaneous saccharification and production of D-LA by *L. bulgaricus* ET45

**Fig. 4:**
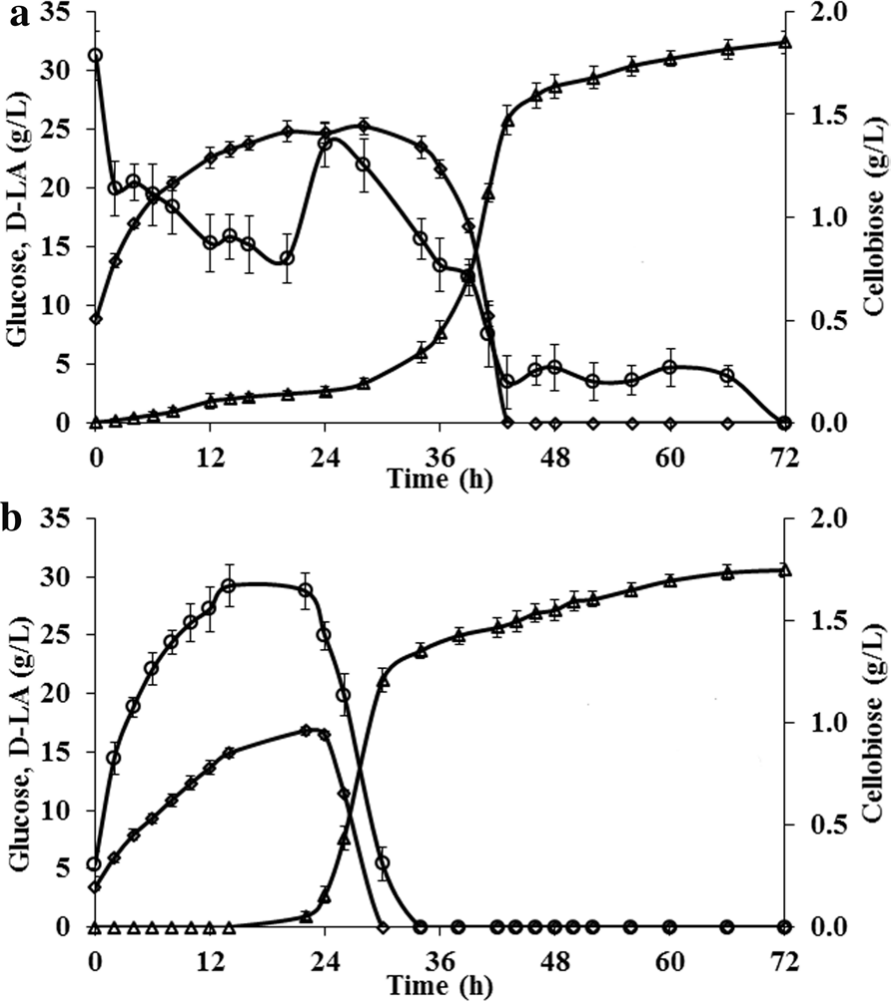
Production of D-LA by *L. bulgaricus* ET45 in batch SSF, Experiments were conducted at 45 °C, pH 5.5 and 10% (w/v) pre-treated LCB (dil. acid pre-treated at 175 °C) using **a** 15 FPU/g_LCB_, **b** 5 FPU/g_LCB_. Diamond—glucose (g/L), triangle—D-LA (g/L), circle—cellobiose (g/L)

The estimated cellulose to glucose conversion (73.7%) in SSF, with 10% (w/v) solid loading and 15 FPU/g_LCB_ at 45 °C, pH 5.5 (Fig. 4a), is substantially higher than that achieved in similar conditions with batch enzyme hydrolysis (60.6%) and also higher than the batch enzymatic hydrolysis at the optimal conditions of 50 °C, pH 4.5 and 15 FPU/g_LCB_ (Table 2).

The higher conversion achieved in SSF is obviously due to the concomitant microbial consumption of glucose which reduces the inhibition on enzymatic hydrolysis. This is also emphasized by the higher hydrolysis efficiency of SSF with the wild-type *L. bulgaricus* WT at 37 °C, pH 5.5 and 5FPU, in comparison with the batch enzyme hydrolysis at the same conditions (Table 2). However, hydrolysis efficiency of SSF by *L. bulgaricus* WT at 37 °C is inferior to the SSF by *L. bulgaricus* ET45 at 45 °C (Table 2).

In earlier study, we have already proved that glucose removal during enzymatic cellulose hydrolysis improves the conversion efficiency, especially during the early part of the hydrolysis (Additional file 1: Fig. S6). This allows for the possibility of reducing the enzyme usage in SSF, since the optimal value of 15 FPU/g_LCB_ for batch enzyme hydrolysis was achieved without removing glucose inhibition. Therefore, we carried out the SSF process (at 45 °C and pH 5.5) at a reduced enzyme loading of 5 FPU/g_LCB_ (Fig. 4b). As can be seen from Table 2, the estimated glucose conversion and the D-LA conversion from cellulose is only marginally (~ 5%) less with an enzyme loading of 5 FPU/g_LCB_ as compared with that obtained at 15 FPU/g_LCB_. Though there is no significant difference in the extent of cellulose hydrolysis between 37 and 45 °C in the later stages of batch enzymatic hydrolysis irrespective of enzyme loading (Additional file 1: Fig S4), we could see a significant increase of ~ 20% in the cellulose to glucose and D-LA conversions by increasing the operating temperature of SSF from 37 to 45 °C at an enzyme loading of 5 FPU/g_LCB_ (Table 2). This difference in cellulose conversions can be attributed to the reduced product inhibition in SSF. These experiments signify the utility of a thermo-tolerant organism in reducing the enzyme loading and maximally exploiting the potential of the enzyme at its near optimal conditions.

To determine the possibility of further reduction in enzyme usage, we carried out the 45 °C SSF experiment with 3 FPU/g_LCB_ (Additional file 1: Fig. S9b). The estimated glucose conversion was ~ 54% and D-LA conversion was ~ 46%, which is much lower than that obtained under the same conditions with SSF at 5 FPU/g_LCB_ (Table 2). Therefore, we carried out further SSF experiments with *L. bulgaricus* ET45 at 5 FPU/g_LCB_, which is much more economical and has only marginally lower conversion than obtained at 15 FPU/g_LCB_ (Fig. 4).

In the batch SSF process, the D-LA accumulation with 10% (w/v) pre-treated LCB loading is only ~ 30 g/L. The difficulty of mixing of pre-treated LCB in reactor precludes the LCB concentrations higher than 10% solid loading. Therefore, we investigated a pulse-fed SSF process, involving intermittent feeding of pre-treated LCB and enzyme addition of 5 FPU/g_LCB_, to enhance D-LA accumulation.

### Pulse-fed SSF for D-LA production

**Fig. 5:**
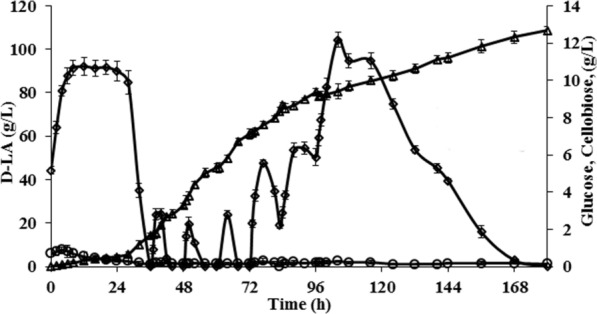
Production of D-LA by *L. bulgaricus* ET45 in pulse fed-batch SSF. Experiment was conducted at 45 °C, pH 5.5 using 5 FPU/g_LCB_ with 35% (w/v) total solid loading (Initial solid loading 5%, pulsed 5% pre-treated LCB (dil. acid pre-treated at 175 °C) and enzyme each at 36th, 48th, 60th, 72nd, 84th and 96th h). Diamond—glucose (g/L), triangle—D-LA (g/L), circle—cellobiose (g/L)

To avoid the difficulty associated with mixing at initial solid loading of 10%, the pulse-fed SSF was started as batch SSF with 5% (w/v) initial solid loading. In the initial 24 h, we could observe accumulation of glucose (Fig. 5) during the lag phase of bacterial growth. After 24 h, the glucose level started to fall with concomitant increase in D-LA accumulation due to the bacterial fermentation. Pulse-feeding of pre-treated LCB (rice straw) and enzyme, at 5% solids (w/v) with 5 FPU/g_LCB_, was started at 36th hour, after the glucose dropped to a low level, and continued till 96th hour. Transient accumulation of glucose was observed after each pulse, due to higher initial rate of hydrolysis than microbial glucose uptake rate. The increased accumulation of glucose in the later stages of pulse-feed can be attributed to lesser glucose uptake rate by the bacteria than the rate of hydrolysis.

The D-LA titer achieved in this pulse-fed SSF was ~ 108 g/L, among the highest reported with SSF processes using pre-treated lignocellulosics as the carbon source (Additional file 1: Table S1). The estimated conversion of cellulose to glucose during pulse-fed SSF was 70.65%, consistent with our results from batch SSFs.

## Discussions

Enzyme costs are a significant fraction of the overall process and product costs in the bioconversion of LCB to bulk chemicals. Usually the bioconversion involves a two-step process of enzymatic hydrolysis of cellulose and fermentation of the sugars in the hydrolysate. This has two drawbacks—lower productivity due to the sequential enzymatic hydrolysis and fermentation processes; and lower conversion of cellulose to glucose due to product inhibition of the enzyme by cellobiose and glucose. The latter problem can be overcome by either continuous removal of glucose [13, 41] or by excessive addition of enzyme, which in turn enhances the process cost. We have demonstrated in this work that SSF is an efficient strategy for enhancing both productivity and conversion while reducing the enzyme usage. This strategy succeeds only when the fermentation process is conducted at conditions closer to the optimal one for enzymatic hydrolysis. Since the hydrolysis is carried out at a moderately high temperature and low pH, it necessitates the use of a thermo-tolerant strain which can perform the fermentation with the same efficiency as at lower temperature.

There are studies which have demonstrated the production of L-LA or LA by thermo-tolerant microbes. A thermo-tolerant *Bacillus licheniformis* TY17 was employed for L-LA production from kitchen refuse in non-sterile condition at 50 °C [42] to achieve 40 g/L of L-LA with a productivity of 2.5 g/L/h. Patel et al. [43] demonstrated the production of L-LA from crystalline cellulose and hemicellulose derived from sugar cane bagasse using a thermo-tolerant acidophilic *Bacillus* sp.at 55 °C and pH 5.0. Hu et al. [15] employed a *Bacillus coagulans* strain for producing L-LA in high titres (97.59 g/L) from alkali pre-treated corn stover by SSF at 50 °C and pH 6.0 with an enzyme loading of 30 FPU/g_LCB_. All of these studies have focused on production of L-LA from agro-industrial or house-hold kitchen waste by employing thermo-tolerant *Bacillus* strains. However, the number of studies on D-LA production by thermo-tolerant microbes in SSF is very limited. In one study [10], an engineered *Pedicoccus acidilactici* was employed at 45 °C for the batch production of D-LA in SSF of LCBs (Additional file 1: Table S1). This was seen to produce a D-LA titre of 76.8 g/L with a D-LA yield of 0.58 from cellulose.

The utility of D-lactate dehydrogenase (D-LDH) of *L. delbrueckii* subsp. *bulgaricus* in high temperature fermentation has been demonstrated by Awasthi et al. [44]. The heterologous expression of D-LDH in an engineered *Bacillus subtilis* resulted in a D-LA titre of 54 g/L from glucose at a yield of 0.89 at 48 °C. In this work, we evolved a thermo-tolerant strain of *L. bulgaricus* ET45, which is capable of growing efficiently at 45 °C and converting glucose in the cellulose hydrolysate to D-LA with the same yield as at 37 °C. This strain was used for SSF processes conducted at 45 °C and pH 5.5. The batch SSF process conducted with 10% LCB loading and 15 FPU/g_LCB_ showed a much higher conversion of cellulose to D-LA (Fig. 4a). At a reduced enzyme loading of 5 FPU/g_LCB_ (Fig. 4b), the conversion was only marginally lower than at 15 FPU/g_LCB_ (Table 2). However, the SSF with 5 FPU/g_LCB_ has nearly 3 times higher product to enzyme ratio (PER) compared to the SSF at 15 FPU/g_LCB_; therefore, this is a worthwhile trade-off in terms of process economics. Since the conversion was much lower for a SSF process with 3 FPU/g_LCB_, we maintained 5 FPU/g_LCB_ as the optimal enzyme loading. The cellulose to D-LA conversion we achieved in batch SSF is comparable to that obtained in a similar process from acid pre-treated corn cob using an enzyme loading of 15 FPU/g_LCB_ and engineered *Pediococcus acidilacti* [10].

It is evident from Additional file 1: Table S1 that the PER that we have achieved (0.062) is comparable or better than many similar studies involving pre-treated LCBs. The higher PERs and D-LA yield reported elsewhere on D-LA production from LCB by SSF have either utilized highly de-lignified biomass (with enhanced cellulose content) [8, 11, 30, 45] or have used sugars from both cellulosic and hemi-cellulosic fractions to achieve higher yields [11, 46]. The higher PERs reported in such studies [8, 30] is not the effect of optimized enzyme addition but can be attributed to the absence of lignin or hemi-cellulosic fractions and enhanced cellulose content obtained by better pre-treatment methods. These studies have reported comparatively higher enzyme loading and lesser solid loading than used in our study.

Batch SSF processes have the disadvantage of limited initial LCB loading due to difficulty in mixing as well as high glucose accumulation before the onset of fermentation, which inhibits the enzymatic hydrolysis. We found it difficult to increase the LCB loading beyond 10% in batch SSF process. This limits the product titer obtained in batch SSF processes. To improve the product titer, we designed a pulse fed-batch SSF process, wherein we pulsed 5% pre-treated LCB with addition of enzyme at 5 FPU/g_LCB_, at 12-h intervals from 36^th^ hour to 96^th^ hour. This process resulted in D-LA titer of ~ 108 g/L, which is among the highest reported with cellulosic biomass. Liu et al. [29] have reported a D-LA titre of 113.18 g/L from whey permeate in fed batch culture which is similar to the titre (108 g/L) we have achieved in the pulse-feed SSF experiment. The D-LA titer and the productivity can be further improved by optimizing the pulse fed-batch SSF process.

One of the important conclusions from the fed-batch work is that addition of LCB in a non-sterile fashion did not lead to contamination or loss of conversion over a 180-h period. This is the advantage of carrying out the SSF at a higher temperature and lower pH. This process thus has significant energy savings over the SHF process which would require sterilization of the cellulosic hydrolysate before fermentation.

The kinetics of hydrolysis and fermentation needs to be modeled and the SSF process can be optimized further in terms of enzyme and LCB feeding rates as well as synchronizing the bacterial growth with the enzymatic hydrolysis. We noticed a prolonged lag phase for the bacterial growth in the SSF process. This could be reduced by initiating the process at lower temperature, when the slower enzyme kinetics would allow for lesser glucose accumulation and then ramping up the temperature slowly to enhance the enzyme kinetics while allowing for bacterial adaptation.

## Conclusions

Development of thermo-tolerant strain of *L. bulgaricus* by evolutionary engineering method allowed us to execute SSF at elevated temperatures and enabled a substantial reduction in enzyme required for production of platform chemicals like D-LA from LCB. This 67% reduction in enzyme usage would definitely improve the economic feasibility of D-LA production from LCB. The D-LA titer (~ 108 g/L) achieved in this work by pulse-feeding of pre-treated rice straw biomass is among the highest demonstrated for production of D-LA from LCB. This study paves the way for development of more thermo-tolerant strains by ALE for the SSF-based conversion of LCB to other platform chemicals.

## Materials and methods

### Microorganism and media

*Lactobacillus bulgaricus* was procured from DSMZ (Germany) and MRS media containing peptone (10 g/L), Beef extract (10 g/L), Yeast extract (5 g/L), Sodium acetate·3H_2_O (5 g/L), Tri-ammonium citrate (2 g/L), K_2_HPO_4_, (2 g/L), MgSO_4_·7H_2_O (0.2 g/L), MnSO_4_·4H_2_O (0.05 g/L), Tween 80 (1 g/L) with 0.5% (w/v) glucose was used for regular cultivation.

### Adaptive laboratory evolution

The experiments were carried out in 250 mL flask containing 100 mL MRS media. The adaptation medium was kept as similar to MRS media, except that 0.7% (w/v) glucose was used. The adaptive evolution was carried out by sequential transfer of culture and growing it with incremental increase in temperature. The subculture was carried out with 10% (v/v) of inoculum taken from mid exponential phase (1.6–1.8 OD) of previous culture. The first seed culture was grown at 37 °C, which is growth optimum temperature for *L*. *bulgaricus* and most of the lactobacilli. The temperature was increased from 37 °C to 40 °C with an increment 1 °C, and then 40 °C to 45 °C with step increments of 0.2 °C. The adaptation experiment was carried out for 68 passages over 116 days.

### Pretreatment of LCB

The rice straw, procured from local market, was air dried and powdered in a domestic grinder to get particle size of < 1 mm. Subsequently, the powdered rice straw biomass was subjected to dilute-acid pretreatment followed by steam explosion. In dilute acid pretreatment, 150 g of dried powdered rice straw was soaked in 2 L of 0.2% (w/v) H_2_SO_4_ for 24 h. The suspension was loaded into a 5 L high pressure vessel (Amar Equipments Pvt. Ltd, Mumbai, India), heated to 175 °C and maintained at 175 ± 5 °C for 30 min followed by decompression to atmospheric pressure by rapid opening of pressure release valve. Pre-treated liquor was removed from treated LCB by centrifugation and dried at 40 °C till constant dry weight was achieved. Cellulose and hemicellulose content of the pre-treated rice straw were determined by two-stage acid hydrolysis as mentioned NREL protocol for the Determination of Structural Carbohydrates and Lignin in Biomass [47]. The virgin rice straw biomass had a glucan and xylan content of 32.66% and 14.68%, respectively. The pre-treated rice-straw with 45.84% cellulose and 4.63% xylan was used for the experiments.

### Enzymatic hydrolysis

All enzymatic hydrolysis experiments were performed using SacchariSEB C6L Plus, a commercial cellulase complex procured from Advanced Enzymes Technology Ltd. (Thane, India). Cellulase activity was measured in FPU as mentioned in the relevant NREL procedure [48]. All the shake flask experiments were carried out with 10% (w/v) of pre-treated LCB. The enzymatic hydrolysis experiments were conducted at different enzyme loadings (5–30 FPU/g_LCB_), temperatures (37 °C, 45 °C and 50 °C) and at two different pH of 4.5 and 5.5. Sodium citrate (0.05 M) was used as buffer for enzymatic hydrolysis and pH was adjusted with 2 N NaOH. Effect of product inhibition was studied by adding different concentrations of glucose (0–60 g/L) or cellobiose (0–5 g/L) along with the pre-treated LCB at 45 °C and pH 5.5 before the initiation of hydrolysis. Percentage cellulose conversion is calculated by the following formula.

% Cellulose to glucose conversion = Glucose (g/L)/(LCB (g/L)*0.46*1.11)*100, where 0.46 is the fraction of cellulose in the pre-treated LCB used and 1.11 (180/162) is the correction factor for accounting the increase in weight of glucose released from cellulose on hydrolysis.

### Batch fermentation of *L. bulgaricus* for D-LA production

Preliminary batch fermentation experiments were conducted using *L. bulgaricus* WT and evolved strain *L. bulgaricus *ET45 in MRS media containing 30 g/L glucose. All fermentation experiments including SHF and SSF were carried out in a 2.5 L INFORS HT Bench-top bioreactor (Infors AG, Bottmingen, Switzerland) with total initial working volume of 1.2 L and 10% inoculum.

### Production of D-LA by SHF

Batch hydrolysis was performed at the optimal conditions of pH 4.5 and 50 °C at 10% (w/v) pre-treated LCB in shake flasks. The hydrolysate was harvested by centrifugation and then sterilized by autoclaving at 10 psi for 10 min. SHF experiments at pH of 5.5 were carried out using *L. bulgaricus* WT and ET45 at 37 °C and 45 °C, respectively. Experiments were initiated in hydrolysate with initial glucose equivalent of 30 g/L in MRS media (except for pure glucose used in batch fermentations). Percentage cellulose to D-LA conversion is calculated by the following formula.

$\% {\text{Cellulose to D - LA conversion}}\, = \,{\text{D - LA }}\left( {{\text{g}}/{\text{L}}} \right)/\left( {{\text{LCB }}\left( {{\text{g}}/{\text{L}}} \right)*0. 4 6* 1. 1 1} \right)* 100$, where 0.46 is the fraction of cellulose in the pre-treated LCB used, 1.11 is the factor for accounting the increase in weight of glucose released from cellulose on hydrolysis.

$\% {\text{Cellulose to glucose conversion in SSF}}\, = \, \% {\text{Cellulose to D - LA conversion}}/0. 8 6$, where 0.86 is Y_D-LA/Glucose_ estimated from SHF experiments.

### Production of D-LA by simultaneous saccharification and fermentation

Required weight (w/v) of pre-treated biomass was sterilized in reactor followed by the feeding of MRS media (except glucose) with working volume of 1.2 L. After feeding of media, required amount of enzyme (FPU/g_LCB_) was added and kept at 300 RPM for hydrolysis. After 1 h of addition of enzyme, 100 mL of inoculum of 1.8 OD (~ 10^10^ cells/100 mL) was added and the fermentation was initiated. Temperature was set at 45 °C and pH was maintained at 5.5 by automatic addition of 4 N KOH. In the fed-batch process, 5% (w/v) non-autoclaved, dry, pre-treated rice straw and enzyme cocktail to maintain total enzyme loading of 5 FPU/g_LCB_ was added every 12 h intervals starting from 36th till 96th hour.

### Quantification of metabolites

The culture supernatant was diluted (20 times) and filtered with a 0.22 μm membrane. Glucose, cellobiose, xylose were estimated by ion-exchange chromatography (IEC) using HPLC (Shimadzu, Japan) equipped with a Phenomenex Rezex 300 × 7.8 mm column and a guard column (35 × 7.8 mm), maintained at 50 °C. Furfural and HMF were estimated by IEC using PDA detector. The mobile phase used for IEC was 5 mM H_2_SO_4_ and flow rate was maintained at 0.6 mL/min. D-LA was estimated using chiral column (Chirex penicillamine-D, Phenomenex India) using 1 mM Cu(II)SO_4_ as mobile phase at 1 mL/min flow rate and 30 °C.

## Supplementary information

**Additional file 1: Table S1.** Comparison of reported titer and yield of D-LA produced from lignocellulosics in literature and current work. **Fig S1.** Growth profile of *L. bulgaricus* WT on 30 g/L glucose at 37 °C in MRS media of a) 6 pH b) 5.5 pH. **Fig S2.** Growth profile of *L. bulgaricus* WT in LCB hydrolysate (SHF) at 37 °C and pH 5.5. **Fig S3.** Effect of temperature on enzymatic cellulose hydrolysis with 10% (w/v) solid loading using 5 FPU/g_LCB_ at pH 5.5. **Fig. S4.** Tukey Simultaneous 95% Confidence Intervals –of one way ANOVA comparing the effect of temperature on enzymatic cellulosic hydrolysis with 10% (w/v) solid loading using at pH 5.5 at enzyme loadings of a) 5 FPU/g_LCB_ and b) 15 FPU/g_LCB_. **Fig S5.** Effect of Cellobiose on cellulose hydrolysis with 10% (w/v) solid loading at pH 5.5 and 15 FPU/g_LCB_. Time course profile of (a) net cellobiose accumulation (b) percentage cellulose conversion. **Fig S6.** Effect of glucose removal on enzymatic cellulose hydrolysis with different enzyme loadings. **Fig. S7.** Growth profile of *L. bulgaricus* WT on 30 g/L glucose at in MRS media of pH 5.5 at a) 40 °C and b) 42 °C. **Fig. S8.** Growth profile of *L. bulgaricus* ET45 at 45 °C and pH 5.5 in a) 30 g/L glucose b) lignocellulosic hydrolysate containing 30 g/L glucose (SHF). **Fig S9.** Production of D-LA in batch SSF at pH 5.5 with 5% (w/v) solid loading by a) *L. bulgaricus* ET45 at 5 FPU/g_LCB_ and 45 °C (b) *L. bulgaricus* ET45 at 3 FPU/g_LCB_ and 45 °C (c) *L. bulgaricus* WT at 5 FPU/g_LCB_ and 37 °C. Values are mean ± SD (n = 3).

## Data Availability

All data generated or analyzed during this study are included in this published article and its additional file 1.

## Abbreviations

µ: Specific growth rate
ALE: Adaptive laboratory evolution
D-LA: D-lactic acid
D-LDH: D-Lactate dehydrogenase
LCB: Lignocellulosic biomass
FPU: Filter Paper Unit
IEC: Ion exchange chromatography
L-LA: L-lactic acid
PER: Product to enzyme ratio
q_s_: Specific glucose uptake rate
SHF: Separate hydrolysis and fermentation
SSF: Simultaneous saccharification and fermentation
WT: Wild type
EH: Enzyme hydrolysis
