# Supplementary material for: Evolutionary engineering of Lactobacillus bulgaricus reduces enzyme usage and enhances conversion of lignocellulosics to D-lactic acid by simultaneous saccharification and fermentation
Source: Biotechnol Biofuels. 2020 Oct 16;13:171. doi: 10.1186/s13068-020-01812-x (PMC7566127; doi:10.1186/s13068-020-01812-x)
Supplement: Supplementary file 1 — Additional file 1: Table S1. Comparison of reported titer and yield of D-LA produced from lignocellulosics in literature and current work. Fig S1. Growth profile of L. bulgaricus WT on 30 g/L glucose at 37 °C in MRS media of a) 6 pH b) 5.5 pH. Fig S2. Growth profile of L. bulgaricus WT in LCB hydrolysate (SHF) at 37 °C and pH 5.5. Fig S3. Effect of temperature on enzymatic cellulose hydrolysis with 10% (w/v) solid loading using 5 FPU/gLCB at pH 5.5. Fig. S4. Tukey Simultaneous 95% Confidence Intervals –of one way ANOVA comparing the effect of temperature on enzymatic cellulosic hydrolysis with 10% (w/v) solid loading using at pH 5.5 at enzyme loadings of a) 5 FPU/gLCB and b) 15 FPU/gLCB. Fig S5. Effect of Cellobiose on cellulose hydrolysis with 10% (w/v) solid loading at pH 5.5 and 15 FPU/gLCB. Time course profile of (a) net cellobiose accumulation (b) percentage cellulose conversion. Fig S6. Effect of glucose removal on enzymatic cellulose hydrolysis with different enzyme loadings. Fig. S7. Growth profile of L. bulgaricus WT on 30 g/L glucose at in MRS media of pH 5.5 at a) 40 °C and b) 42 °C. Fig. S8. Growth profile of L. bulgaricus ET45 at 45 °C and pH 5.5 in a) 30 g/L glucose b) lignocellulosic hydrolysate containing 30 g/L glucose (SHF). Fig S9. Production of D-LA in batch SSF at pH 5.5 with 5% (w/v) solid loading by a) L. bulgaricus ET45 at 5 FPU/gLCB and 45 °C (b) L. bulgaricus ET45 at 3 FPU/gLCB and 45 °C (c) L. bulgaricus WT at 5 FPU/gLCB and 37 °C. Values are mean ± SD (n = 3). [file 13068_2020_1812_MOESM1_ESM.docx]

**Additional file 1**

**Evolutionary engineering of *Lactobacillus bulgaricus* reduces enzyme usage and enhances conversion of lignocellulosics to D-lactic acid by simultaneous saccharification and fermentation**

Vishnu Prasad J^1#^, Tridweep K. Sahoo^1#^, Naveen S., Guhan Jayaraman^1*^

^1^Bioprocess and Metabolic Engineering Laboratory,

Department of Biotechnology, Bhupat and Jyoti Mehta School of Biosciences,

Indian Institute of Technology-Madras, Chennai, Tamil Nadu, India, 600036

^#^ These authors have contributed equally to the work.

^*^Corresponding author: Guhan Jayaraman

email id: guhanj@iitm.ac.in

**Table S1.** Comparison of titer and yield of D-LA among previously reported literature

**Figure S1.** Growth profile of *L. bulgaricus* WT on 30 g/L glucose at 37 °C in MRS media of a) 6 pH b) 5.5 pH . Values are mean ± SD (n=3).

**Figure S2.** Growth profile of *L. bulgaricus* WT in LCB hydrolysate (SHF) at 37 °C and pH 5.5. Values are mean ± SD (n=3).

**Figure S3.** Effect of temperature on enzymatic cellulose hydrolysis with 10% (w/v) solid loading using 5 FPU/g_LCB_ at pH 5.5. Values are mean ± SD (n=3).

**Figure S4.** Tukey Simultaneous 95% Confidence Intervals –of one way ANOVA comparing the effect of temperature on enzymatic cellulosic hydrolysis with 10% (w/v) solid loading using at pH 5.5 at enzyme loadings of a) 5 FPU/g_LCB_ and b) 15 FPU/g_LCB_. Values are mean ± SD (n=3).

**Figure S5.** Effect of Cellobiose on cellulose hydrolysis with 10% (w/v) solid loading at pH 5.5 and 15 FPU/g_LCB._ Time course profile of (a) net cellobiose accumulation (b) percentage cellulose conversion. Values are mean ± SD (n=3).

**Figure S6.** Effect of glucose removal on enzymatic cellulose hydrolysis with different enzyme loadings. Values are mean ± SD (n=3).

**Figure S7.** Growth profile of *L. bulgaricus* WT on 30 g/L glucose at in MRS media of pH 5.5 at a) 40 °C and b) 42 °C . Values are mean ± SD (n=3).

**Figure S8.** Growth profile of *L. bulgaricus* ET45 at 45 °C and pH 5.5 in a) 30 g/L glucose b) lignocellulosic hydrolysate containing 30 g/L glucose (SHF). Values are mean ± SD (n=3).

**Figure S9**. Production D-LA in batch SSF at pH 5.5 with 5 % (w/v) solid loading by a) *L. bulgaricus* ET45 at 5 FPU/g_LCB_ and 45 °C (b) *L. bulgaricus* ET45 at 3 FPU/g_LCB_ and 45 °C (c) *L. bulgaricus* WT at 5 FPU/g_LCB_ and 37 °C. Values are mean ± SD (n=3).

**Table S1:** Comparison of reported titer and yield of D-LA produced from LCB in literature and current work

| Strain | Method | Substrate | Yield (g/g) | Titer (g/L) | Conditions^##^ | PER (g/FPU) | Reference |
| --- | --- | --- | --- | --- | --- | --- | --- |
| *Lactobacillus coryniformis* subsp.*torquens* ATCC 25600 | Batch | Filter paper | 0.89 | ~25 | 39 °C/ pH 5.4  33.33 g/L  28 FPU | 0.027 | Yanez et al. 2003 [45] |
| *Lactobacillus coryniformis* ssp *torquens* ATCC 25600 | Batch | Alkali treated waste cardboard | 0.68 | 13.9 | 39 °C/ pH 5.4  33.33 g/L  28 FPU | 0.015 | Yanez et al. 2005 [7] |
|  | Fed Batch |  | 0.57 | 23.4 | 39 °C/ pH 5.4  66.66 g/L  14 FPU | 0.025 |  |
| *Lactobacillus lactis* mutant RM2-24 | Batch | Alpha cellulose (from sugarcane bagasse) | 0.73 | 73 | 42 °C/ pH 6.5  100 g/L  10 FPU | 0.073 | Singhvi et al. 2010 [8] |
| *L. delbrueckii* ATCC 9649 | Batch | Pulp | 0.48^#^ | 19.2 | 40 °C/ pH 5.5  40 g/L  8 FPU | 0.06 | Zhang and Vadlani 2013 [9] |
|  |  | Alkali pretreated corn Stover | 0.50^#^ | 20.1 |  | 0.063 |  |
| *L. plantarum* NCIMB 8826 ΔldhL1::PxylAB-Δxpk1::tkt-Δxpk2::PxylAB | Batch | Delignified hardwood pulp | 0.88^*^ | 122.8 | 37 °C/ pH 6  150 g/L  20 FPU | 0.041 | Hama et al. 2015 [46] |
| *Pediococcus acidilactici* zp26 | Batch | Dilute acid pretreated corn stover | 0.58 | 76.8 | 45 °C/ pH 5.5  25% (w/w)  15 FPU | 0.015 | Yi et al. 2016 [10] |
| *L. plantarum* NCIMB  8826 ΔldhL1-pLEM415-xylAB | Batch | Alkali pretreated corn Stover | 0.73^*#^ | 29.4 | 37 °C/ pH 5.5  40 g/L  8 FPU | 0.092 | Zhang et al 2016 [11] |
|  | Fed-batch |  | 0.77^*#^ | 61.4 | 45 °C  80 g/L  5.6 FPU | 0.137 |  |
| *L. bulgaricus* | Batch | Organosolv pretreated beech wood | 0.69^#^ | 62 | 44 °C/pH 5.5  90 g/L  9.6 FPU | 0.072 | Karnaouriet al 2020 [30] |
| ***L. bulgaricus* ET45** | **Fed- batch** | **Dilute acid pretreated rice straw** | **0.67** | **108.6** | **45 °C/pH 5.5**  **350 g/L**  **5 FPU** | **0.062** | **This study** |

*D-LA is formed from both glucose and xylose
Yield is calculated by the amount of D-LA divided by the amount of cellulose present in the biomass pretreated LCB
^#^product overall yield was calculated by the amount of D-lactic acid produced divided by the total amount of LCB used
##Indicates the experimental conditions in the order of Temperature, pH, Biomass loading and Enzyme loading in FPU per gram of LCB; PER is the amount of D-LA produced in grams per FPU of enzyme.

**
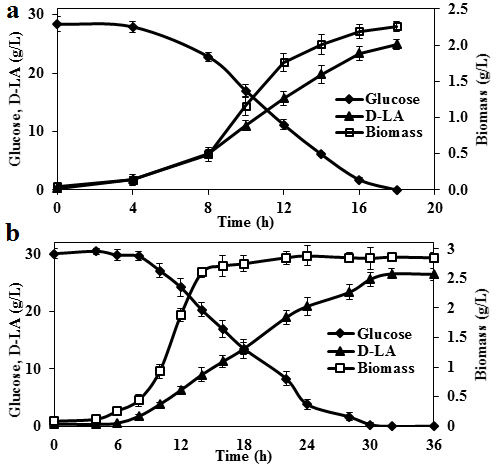
**

**Figure S1.** Growth profile of *L. bulgaricus* WT on 30 g/L glucose at 37 °C in MRS media of a) 6 pH b) 5.5 pH. Values are mean ± SD (n=3).

**
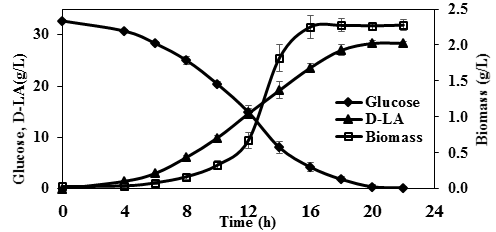
**

**Figure S2.** Growth profile of *L. bulgaricus* WT in LCB hydrolysate (SHF) at 37 °C and pH 5.5. Values are mean ± SD (n=3).


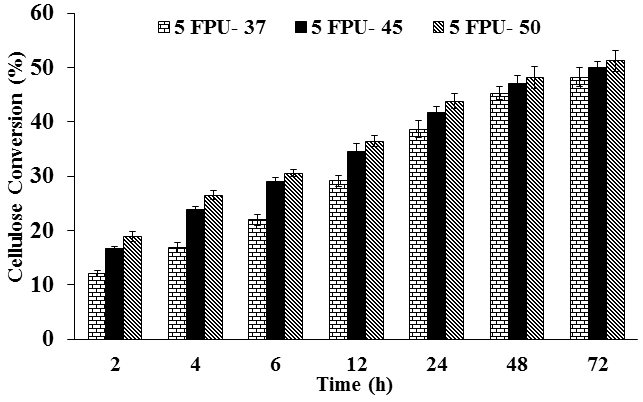


Figure S3: Effect of temperature on enzymatic cellulose hydrolysis with 10% (w/v) solid loading using 5 FPU/g_LCB_ at pH 5.5. FPU-37 denotes the corresponding enzyme loading (5 FPU/g_LCB_) at 37 °C, FPU- 45 denotes the corresponding enzyme loading (5 FPU/g_LCB_) at 45 °C, FPU-50 denotes the corresponding enzyme loading (5 FPU/g_LCB_) at 50 °C. Values are mean ± SD (n=3).

**
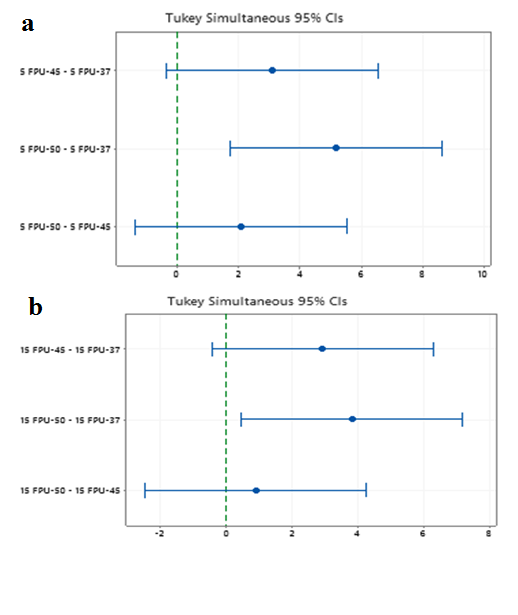
**

Figure S4: Tukey Simultaneous 95% Confidence Intervals –of one way ANOVA comparing the effect of temperature on enzymatic cellulosic hydrolysis with 10% (w/v) solid loading using at pH 5.5 at enzyme loadings of a) 5 FPU/g_LCB_ and b) 15 FPU/g_LCB_. The plot compares the means between the three operating temperatures (If an interval does not contain zero, the corresponding means are significantly different.).

**
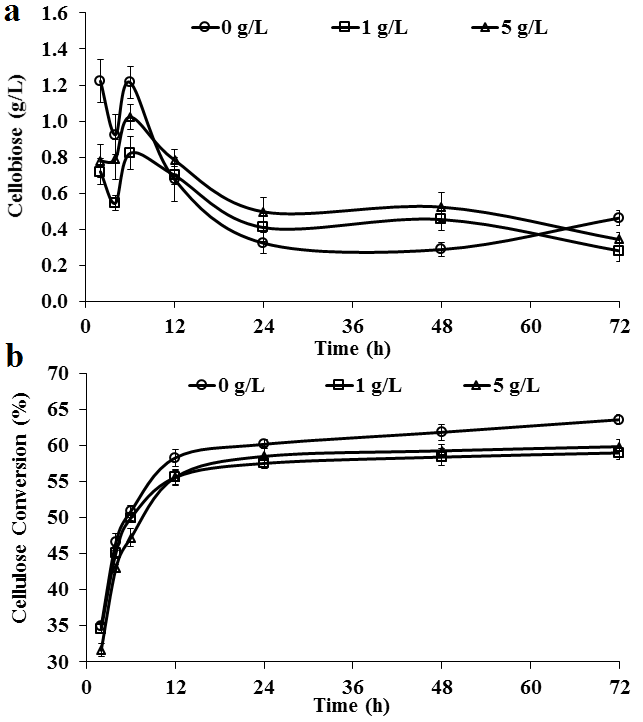
**

Figure S5: Effect of Cellobiose on cellulose hydrolysis with 10% (w/v) solid loading at pH 5.5 and 15 FPU/g_LCB._ Time course profile of (a) net cellobiose accumulation (b) percentage cellulose conversion. Values are mean ± SD (n=3).


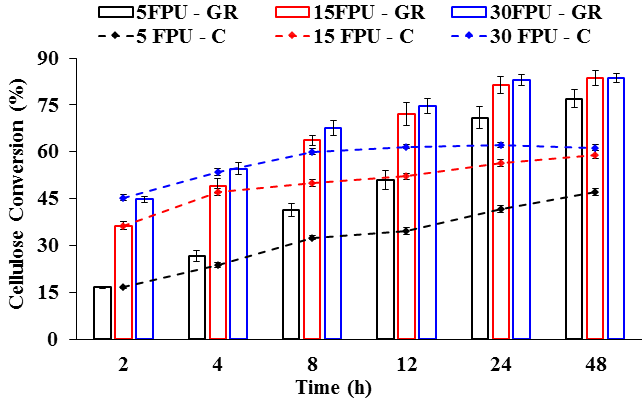


Figure S6: Effect of glucose removal on enzymatic cellulose hydrolysis with different enzyme loadings (5, 15, 30 FPU/g_LCB_) using 10% (w/v) solid loading at pH 5.5 and temperature 45 °C. FPU-GR denotes the corresponding enzyme loading (FPU/g_LCB_) with glucose removal, FPU-C denotes the corresponding enzyme loading (FPU/g_LCB_) without glucose removal (control). Values are mean ± SD (n=3).


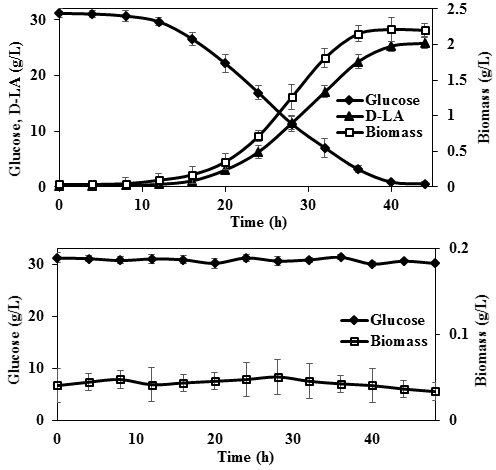


**Figure S7.** Growth profile of *L. bulgaricus* WT on 30 g/L glucose at in MRS media of pH 5.5 at a) 40 °C and b) 42 °C. Values are mean ± SD (n=3).


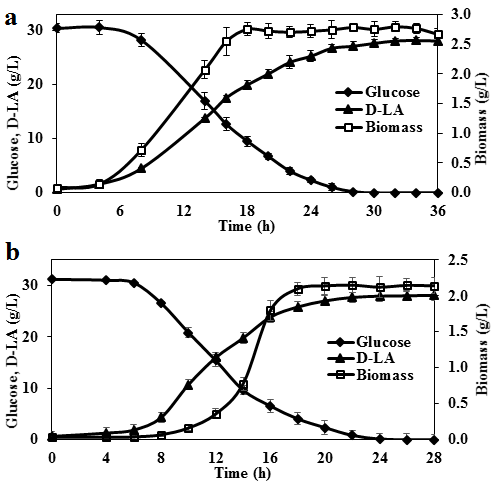


**Figure S8.** Growth profile of *L. bulgaricus* ET45 at 45 °C and pH 5.5 in a) 30 g/L glucose b) lignocellulosic hydrolysate containing 30 g/L glucose (SHF). Values are mean ± SD (n=3).


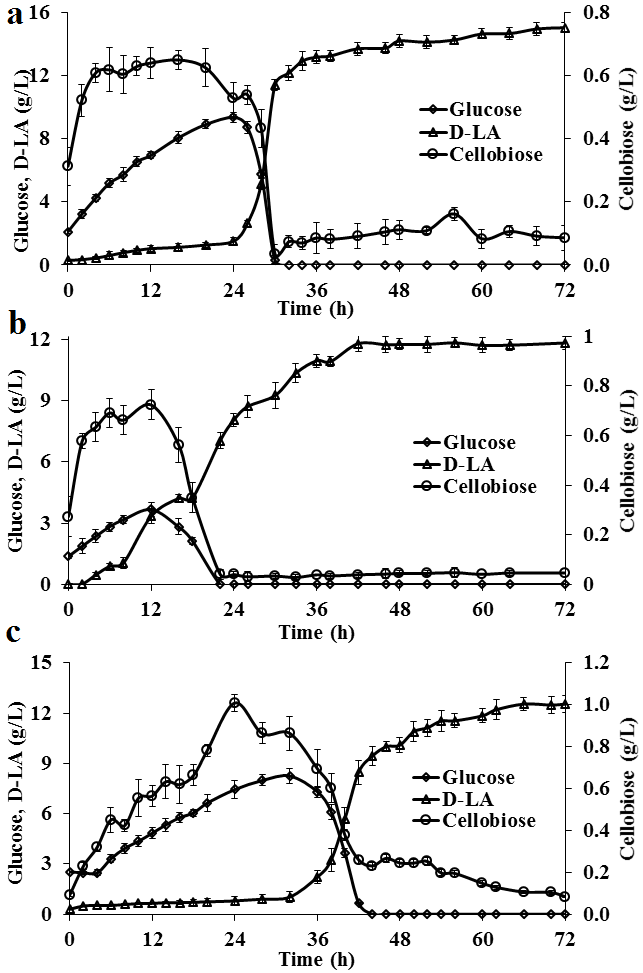


Figure S9: Production D-LA in batch SSF at pH 5.5 with 5 % (w/v) solid loading a) *L. bulgaricus* ET45 at 5 FPU/g_LCB_ and 45 °C (b) *L. bulgaricus* ET45 at 3 FPU/g_LCB_ and 45 °C (c) *L. bulgaricus* WT at 5 FPU/g_LCB_ and 37 °C. Values are mean ± SD (n=3).
